# Implementing Precision Antimicrobial Therapy for the Treatment of Bovine Respiratory Disease: Current Limitations and Perspectives

**DOI:** 10.3389/fvets.2017.00143

**Published:** 2017-08-29

**Authors:** Guillaume Lhermie, Pierre-Louis Toutain, Farid El Garch, Alain Bousquet-Mélou, Sébastien Assié

**Affiliations:** ^1^Department of Population Medicine and Diagnostic Science, College of Veterinary Medicine, Cornell University, Ithaca, NY, United States; ^2^INRA, UMR1331 TOXALIM, Toulouse, France; ^3^Université de Toulouse, INPT, ENVT, EIP, UPS, Toulouse, France; ^4^Vétoquinol, Global Drug Development, Lure, France; ^5^INRA, UMR1300 Biologie, Epidémiologie et Analyse de Risque en santé animale BioEpAR, Nantes, France

**Keywords:** antimicrobial use, decreased regimen, early treatment, fluoroquinolone, cattle, bovine respiratory disease complex, veterinary precision medicine

## Abstract

The therapeutic efficacy of an early treatment protocol with an infection-stage adjusted fluoroquinolone regimen was evaluated in a field study on young bulls (YBs) presenting signs of bovine respiratory disease (BRD). A total of 195 YB (Charolais, Limousin, and Rouge-des-Prés breeds) from 6 farms implementing or not prophylactic antimicrobial treatments (PROPHY or absence) were randomly assigned to 1 of 2 experiment groups based on time of detection of BRD and first-line marbofloxacin regimen, early adjusted dose [Early 2 (E2)] or late standard dose [Late 10 (L10)]. Each YB was administered orally a reticulo-rumen bolus, allowing continuous monitoring of ruminal temperature. In the E2 group, YB presenting early signs of BRD, i.e., an increase in ruminal temperature over 40.2°C and persisting more than 12 h, confirmed by a clinical examination showing no or mild signs of BRD, were given 2 mg/kg of marbofloxacin. In the L10 group, YBs presenting moderate or severe signs of BRD at visual inspection, confirmed at clinical examination, were given 10 mg/kg of marbofloxacin. If needed, YBs were given a relapse treatment. The YBs were followed for 30 days. The proportions of first and relapse treatments were calculated, as well as the therapeutic efficacy at day 10. In the E2 group, the first-line treatments’ proportion was significantly higher (*P* < 0.05), while the relapse treatments’ proportion tended to be higher (*P* = 0.08), than in the L10 group. Evolution of clinical scores (CSs) of diseased YB was followed for 10 days. In both groups, CS and rectal temperature decreased significantly 24 h after treatment (*P* < 0.05). Treatment incidences (TI) representing antimicrobial consumption assessed on used daily doses (UDD) were calculated. Antimicrobial consumption of marbofloxacin and relapse treatments were not significantly different between the groups. These values were strongly influenced by the recourse to a prophylactic antimicrobial treatment, accounting for more than 90% of the antimicrobial amount in the herds implementing prophylaxis. The higher number of treatments in the groups treated on the basis of ruminal temperature monitoring, the accuracy of the detection method, and the necessary conditions to implement precision antimicrobial therapy in the field are discussed in this article.

## Introduction

Antimicrobial use (AMU) in veterinary medicine may lead to the selection of resistant bacteria, potentially transferred to humans, representing a public health hazard (1, 2). Given the necessity of maintaining available antimicrobials, the context of sustainable use—as little as possible, and as much as necessary—represents a driver for new approaches of antimicrobial therapy in veterinary medicine (3–5).

In food producing animals, the treatment of bovine respiratory diseases (BRD) represents one of the major uses of antimicrobials (6). Because of the high prevalence of bacteria as involved pathogens, treatment of BRD generally includes an empirical antimicrobial drug (AMD) therapy, applied with a wide variety of classes; among them the most frequently used are penicillins, tetracyclines, macrolides, and quinolones (7). Current practices of treatment of BRD consist of (i) treating the entire cohort of animals before the onset of BRD (prophylactic treatment) and (ii) treating the entire cohort of animals in which only a small number of animals expresses clinical signs (metaphylactic treatment or control) (8). The advantages of these mass medication strategies are the control of the infection dissemination and a good survival rate with regards to the group, promoting the prevention and/or delay of BRD in the group. However, such strategies also have the disadvantage of subjecting a potentially large proportion of animals to AMDs potentially able to contaminate the food chain and/or the environment with antimicrobial resistance (AMR) determinants (bacteria and genes). The other therapeutic approach to treat BRD focuses on (iii) curative treatments of only the animals presenting clinical signs of illness (9–11). This approach limits the number of animals exposed to antimicrobials. However, it is usually seen as the “late approach,” and delay in treatment initiation may impact animal welfare, as the clinical signs possibly reflect extensive pulmonary damage which had time to develop prior to treatment (6). Furthermore, in this late approach, dosages of antimicrobials needed to achieve bacterial cure are generally high. Considering the uncertainty regarding the causal agent and time of disease onset, labeled doses are determined by pharmacokinetic/pharmacodynamic (PK/PD) and dose determination experiments. These doses generally target a cluster of pathogens and consider the highest minimal inhibiting concentration reported (12, 13). These conditions lead then to routine use of a “high” antimicrobial dose.

Recent studies have stressed the interests of early therapeutic interventions, when the infectious bacterial load is still low, suggesting that the dose of antibiotics may be modulated concurrently with the infectious bacterial load. A relation between the size of bacterial inoculum and the antibiotic dose needed to achieve bacterial elimination was first discovered by Eagle in 1949 (14). Since then, this so-called “Eagle effect” or “inoculum effect” has been observed in experimental models of infections, showing that the antimicrobial activity of fluoroquinolones or beta-lactams, including third-generation cephalosporins, could be reduced against high bacterial loads compared to low bacterial loads of different bacterial species, including *Escherichia coli, Pasteurella multocida, Klebsiella pneumoniae*, and *Staphylococcus aureus* (12, 13, 15–17). In a calf experimental model of BRD, we observed high bactericidal activity of an adjusted marbofloxacin dose administered “early” on low inoculum of *Mannheimia haemolytica* compared with a late administration, even at a fivefold higher dose (18). However, the lack of diagnostic tools allowing identification of pathogens and bacterial load in field conditions constitutes a major barrier to implement such an early treatment strategy. An alternative could be to monitor early signs of BRD, such as increase in core body temperature, which has been demonstrated to appear before clinical signs (19, 20). Combining appropriate doses of AMD and early detection of BRD could thus result in a reduction in AMU.

The objective of our study was to assess in a field experiment the therapeutic effectiveness of an infection-stage adjusted antimicrobial therapy given at the early stage of BRD, compared to a standard therapy, i.e., a single injection of 10 mg/kg marbofloxacin administered after observation of clinical signs, as recommended by the manufacturer.

## Materials and Methods

### Study Design

The research protocol was compliant with the European Guideline on Good Clinical Practice (CVMP/VICH/595/98-FINAL) and has been submitted to the French National Food Safety Agency, under the reference ENR/KLD/EC-00710-0. All procedures were performed in accordance with the European Directive (2010/63/EU) and French Regulations (Decree no. 2013-118; articles R214-87 and R214-137 from Rural Code) related to the Care and Use of Laboratory Animals. However, because all the procedures involving animals were conducted in commercial farms, and therefore do not fall under the scope of the European Directive (2010/63/EU) on the protection of animals used for scientific purposes (article 1-5), ethics committees established under the French Regulations do not examine approvals of such protocols. The study was conducted in western France from December 2014 to March 2015. In 6 commercial fattening units, 195 ruminant young bulls (YBs) of Charolais, Limousin, and Rouge-des-Prés breeds, aged between 7 and 10 months and with an average body weight of 299 kg were recruited (Table 1). Before their arrival at the fattening unit, YBs were transported over a distance less than 500 km from their birth farm to the sorting facility place. YBs were given eprinomectin Pour-On (Eprinex, Merial, Villeurbanne, France) and were also administered orally a reticulo-rumen temperature bolus (San’phone, Medria SAS, Chateaugiron, France). YBs were then transported by truck for travel distance less than 50 km to the fattening unit. At the time of arrival, YBs were separated into pens containing 7–12 animals. In the six fattening units, each pen was randomly assigned to one of the two experiment groups, Early 2 (E2) and Late 10 (L10), characterized by the different methods of detection of BRD and curative antimicrobials treatment regimens. In 3 fattening units, considered at high risk of BRD occurrence, a prophylactic antimicrobial treatment (PROPHY) was administered to each YB, at the sorting facility. Overall, four groups of animals were studied: PROPHY-E2, PROPHY-L10, E2, and L10. YBs were housed in straw-bedded barns facilities and had free access to water and food. Enrollment of the YB started on the first day on feed and lasted 30 days.

**Table 1:** Characteristics of the herds, number of young bulls (YBs) per group, and proportion of antimicrobial treatments.

Group	Herd	Mean live weight at arrival (kg ± SD)	Antibiotic used for relapse	Number of YBs	Number of bulls with 1 curative treatment	Number of bulls with 2 curative treatments		Proportion of first-line treatment	Proportion of relapse treatment
PROPHY-E2	A	343 ± 19	Flor	10	4	2		60	20
	B	278 ± 30	Flor	21	10	4		67	19
	C	361 ± 21	Flor	20	9	1		50	5
							Mean	59	15
							SD	6	6
PROPHY-L10	A	349 ± 21	Flor	20	5	1		30	5
	B	293 ± 45	Flor	35	2	0		6	0
	C	364 ± 18	Flor	24	3	0		13	0
							Mean	16	2
							SD	9	2
E2	D	286 ± 11	Tula	14	8	3		79	21
	E	248 ± 10	Tula	6	2	1		50	17
	F	273 ± 11	Tula	13	4	2		46	15
							Mean	58	18
							SD	14	2
L10	D	281 ± 16	Tula	7	3	0		43	0
	E	232 ± 21	Tula	15	10	1		73	7
	F	262 ± 9	Tula	8	0	0		0	0
							Mean	39	2
							SD	26	3

*Two out of the 195 YBs were excluded from the study, one for severe signs of lameness 3 days after the start of the study and another with abnormal behavior. In the E2 groups, YBs were given 2 mg/kg of marbofloxacin; in the L10 groups, YBs were given 10 mg/kg of marbofloxacin; YBs from PROPHY-E2 and PROPHY-L10 were given in addition 4 mg/kg bw of tildipirosin at the sorting facility*.

*Flor, florfenicol; Tula, tulathromycin; E2, Early 2; L10, Late 10*.

### BRD Detection and Inclusion Criteria

In the L10 groups, detection of BRD in YB was only based on visual inspection of undisturbed animals followed by clinical examination in case of discomfort signs. A validated grid developed by Torres et al. (21) to perform visual inspection was used. Briefly, a YB presenting clinical signs of BRD after a veterinary physical examination, such as moderate or severe signs of depression, nasal discharge, cough, and increased respiratory efforts at visual inspection, was considered as late detected if also presenting a rectal temperature ≥39.7°C, and included in the study.

In the E2 groups, BRD detection was based on the combination of ruminal temperature continuous monitoring and veterinary clinical examination. Ruminal temperatures of YB were recorded for 30 days after their arrival at the farm, as described by Timsit et al. (20). Briefly, the rumen temperature was recorded every 5 min by the temperature bolus. Data extracted were analyzed by the program provided by the manufacturer and represented graphically by a curve showing ruminal temperature as function of time. The curves were observed three times daily by a veterinarian. If an increase of ruminal temperature over a threshold of 40.2°C and persisting more than 12 h was observed, a clinical examination of the suspected animal was performed within 12 h. Upon examination, YB with a rectal temperature ≥39.7°C and no or mild other clinical signs of BRD (normal demeanor; respiratory rate <60; heart rate <100, no or mild nasal discharge; normal respiration; normal or slightly decreased appetite) were considered as early detected and included. A YB with a rectal temperature <39.7°C at the time of examination was considered as not detected.

Young bulls presenting clinical signs differing from respiratory disease signs (e.g., lameness and diarrhea) were excluded from the study.

### Clinical Follow-up

In case of detection of disease, rectal temperature of the YB was recorded, and a complete clinical examination was performed at the time of first-line treatment (day 0) and at days 1, 2, 3, 7, 10, and 21 after treatment. YBs were monitored using a scoring system adapted from Dowling et al. (22). Scores were assigned as follows: demeanor from 0 to 3 (normal, dull, depressed, and recumbent); rectal temperature from 0 to 2 (<39.5, 39.5–40.5, >40.5); respiratory rate from 0 to 3 (<45, 45–<60, 60–90, >90); heart rate from 0 to 2 (<80, 80–100, >100); nasal discharge from 0 to 3 (absent, mild, moderate, and profuse); respiration from 0 to 2 (normal, increased effort, and labored); appetite from 0 to 2 (normal, decrease, and anorexic). The clinical score (CS) was calculated daily for each YB by adding the values given for each parameter.

At days 1, 2, and 3, YBs presenting an increase in their CS compared to day 0 or an increase >0.5°C in their rectal temperature measured at ay 0 were given a relapse treatment (see later). From day 4 to the end of the study, a YB already treated and presenting moderate to severe clinical signs of BRD was also given a relapse treatment.

Evolution of CS of diseased YB was followed for 10°days and evaluated as a function of time.

### Antimicrobial Treatments

#### Prophylactic Treatments

A prophylactic subcutaneous treatment of 4 mg/kg body weight tildipirosin (Zuprevo, MSD, Beaucouze, France) was administered at the auction market place the day before entering the farm to each YB from herds A, B, and C (PROPHY). In YB from herds D, E, and F, no prophylactic treatment was implemented.

#### First-line Treatments

In the L10 groups, animals identified after clinical examination as late detected were immediately given a single intramuscular dose of 10 mg/kg marbofloxacin (Forcyl, Vetoquinol, Lure, France), as recommended in the summary of product characteristics (SPC). For welfare issues, a single intramuscular dose of 2 mg/kg tolfenamic acid, a non-steroidal anti-inflammatory drug (Tolfine, Vetoquinol, Lure, France), was simultaneously administered. It was shown in ruminants that a simultaneous injection of tolfenamic acid did not affect the marbofloxacin pharmacokinetic profile (23).

In the E2 groups, animals identified after clinical examination as early detected were immediately given a single intramuscular dose of 2 mg/kg marbofloxacin (Marbocyl, Vetoquinol, Lure, France) and a single intramuscular dose of 2 mg/kg tolfenamic acid.

#### Relapse Treatments

During the study period, YB received when needed a relapse treatment. According to the farmers’ customary practices, the relapse treatment was a single subcutaneous injection of 40 mg/kg of florfenicol (Fenflor, Vetoquinol, Lure, France) in herds A, B, and C and a single subcutaneous injection of 2.5 mg/kg of tulathromycin (Draxxin, Zoetis, Malakoff, France) in herds D, E, and F.

In each fattening unit and each group, the proportions of curative first-line and relapse treatments were calculated as the number of YB treated with a first-line and relapse treatment divided by the number of YB in the group, and expressed as a percentage.

### Processing of Antimicrobial Consumption Evaluation

Antimicrobial consumption was calculated using treatment incidences (TI_UDD_) (a scalar) based on used daily dose (UDD) per YB (UDD_YB_) of prophylactic treatment (UDD_tildipirosin_), first-line treatment (UDD_marbofloxacin_) and relapse treatment (UDD_relapse_ = UDD_tulathromycin_ or UDD_florfenicol_) using the following formula:

$${\text{TI}}_{\text{UDD}\left(x\right)}=\frac{\text{Total amount}(\text{mg})\text{ consumed for a given AMD}(x)}{\begin{array}{l} \frac{\text{UDD}(x)\text{ in mg}/\text{kg}}{\text{Corrective factor for }x\text{ in days}}\times \text{ Total kg bw of YBs at risk }\times \text{ Observation period in days} \end{array}},$$

where TI_UDD(_*_x_*_)_ is the TI_UDD_ for a given antimicrobial (AMD) *x*, i.e., tildipirosin, marbofloxacin, florfenicol, or tulathromycin, Total amount (mg) consumed for a given AMD(*x*) is the total amount (in mg) of a given AMD for a given subgroup of YB. UDD was defined as the administered dose in milligrams per day per kilogram of body weight. For marbofloxacin, the retained value of UDD_marbo/YB_ was 2 mg/kg; for tulathromycin, the value of UDD_tula/YB_ was 2.5 mg/kg; for florfenicol, the value of UDD_flor/YB_ was 40 mg/kg; for tildipirosin, the value of UDD_til/YB_ was 4 mg/kg. To account for the long acting formulation of some drugs, a corrective factor, corresponding to the estimated duration of effects (in days), was attributed to each drug, according to references already used by authorities (24, 25). The corrective factors, expressed in days, were 1, 9.3, 7, and 2 for marbofloxacin, tildipirosin, tulathromycin, and florfenicol, respectively. Therefore, the value of UDD (in mg/kg) was divided by the corrective factor. The results were expressed in TI_UDD_ for a YB of 300 kg at risk, representing a YB belonging to a group exposed to BRD in each pen, followed during the observation period of 30 days.

### Statistical Methods

At the farm level, the effects of first and relapse treatments were analyzed using a mixed logistic model with group nested in herd as a random effect. TI_UDD marbo_ and TI_UDD relapse_ were analyzed using a chi-square test for independence. Significance was set at *P* ≤ 0.05. At the individual animal level, for the comparison of CS and rectal temperatures before treatment and for the next 10 days, mixed linear models were tested including the variables group of treatment and time as fixed effects and farm and YB nested in farm as random effects. A similar mixed linear model was built for the comparison of rectal temperature. Statistical analyses were performed with the software SAS (SAS version 9.1 Inst. Inc., Cary, NC, USA).

## Results

### Proportion of Curative Treatments

**Figure 1:**
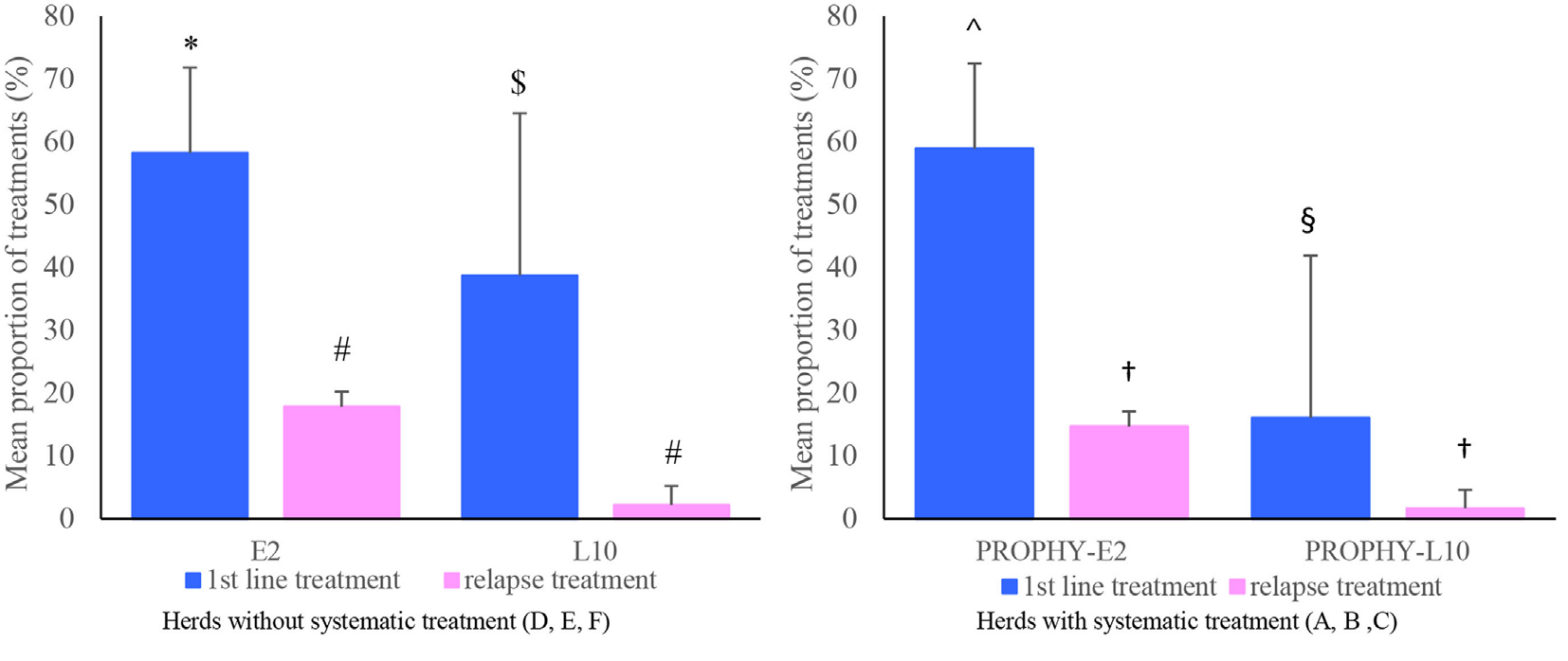
Mean proportion of first and relapse treatments in Early 2 (E2) and Late 10 (L10) groups, according to the prophylactic strategy implemented in the herds (absence or presence). In the E2 groups, young bulls (YBs) were given 2 mg/kg of marbofloxacin; in the L10 groups, YBs were given 10 mg/kg of marbofloxacin; YBs from PROPHY-E2 and PROPHY-L10 were given in addition 4 mg/kg bw of tildipirosin at the sorting facility. The proportion (expressed in percentage) of first-line treatments is calculated as the number of YB treated with a first-line treatment in the group/number of YB in the group. The proportion of relapse treatments is calculated as number of YB treated with a relapse treatment in the group/number of YB in the group. Different signs in superscripts indicate if values are statistically different (*P* < 0.05). No comparison was made between the groups with and without prophylactic treatment.

Among the 195 YB included, 1 YB presenting severe signs of lameness 3 days after the start of the study and 1 YB with abnormal behavior were excluded. Treatments were administered between 2 and 14 days after entering the farm. The proportions of curative treatments are presented in Figure 1. In the herds without prophylactic treatment, the mean proportion of first and relapse treatments were, respectively, 58 ± 14 and 18 ± 2% in the E2 groups. In the L10 groups, the mean proportion of first and relapse treatments was, respectively, 39 ± 26 and 2 ± 3%. In the PROPHY-E2 groups, the mean proportion of first and relapse treatments was, respectively, 59 ± 6 and 15 ± 6%. In the PROPHY-L10 groups, the mean proportion of first and relapse treatments was, respectively, 16 ± 9 and 2 ± 2%. The proportion of first-line treatments was significantly higher (*P* < 0.05) in the E2 groups compared to the L10 groups, regardless of the implementation of prophylactic treatment. The proportion of relapse treatments tended to be higher (*P* = 0.08) in the E2 groups compared to the L10 groups. We observed a strong variability between herds. Overall, herds that did not implement prophylactic antimicrobial treatment had a slightly higher proportion of first and relapse treatments compared to those with such implementation (see Figure 1).

One YB in the E2 group from herd A which received a relapse treatment died after the end of the study. Necropsy results reported severe lesions of suppurative cranial bronchopneumonia, caudal emphysema, and fibrino-hemorragic pleuritis.

### Clinical Scores

**Figure 2:**
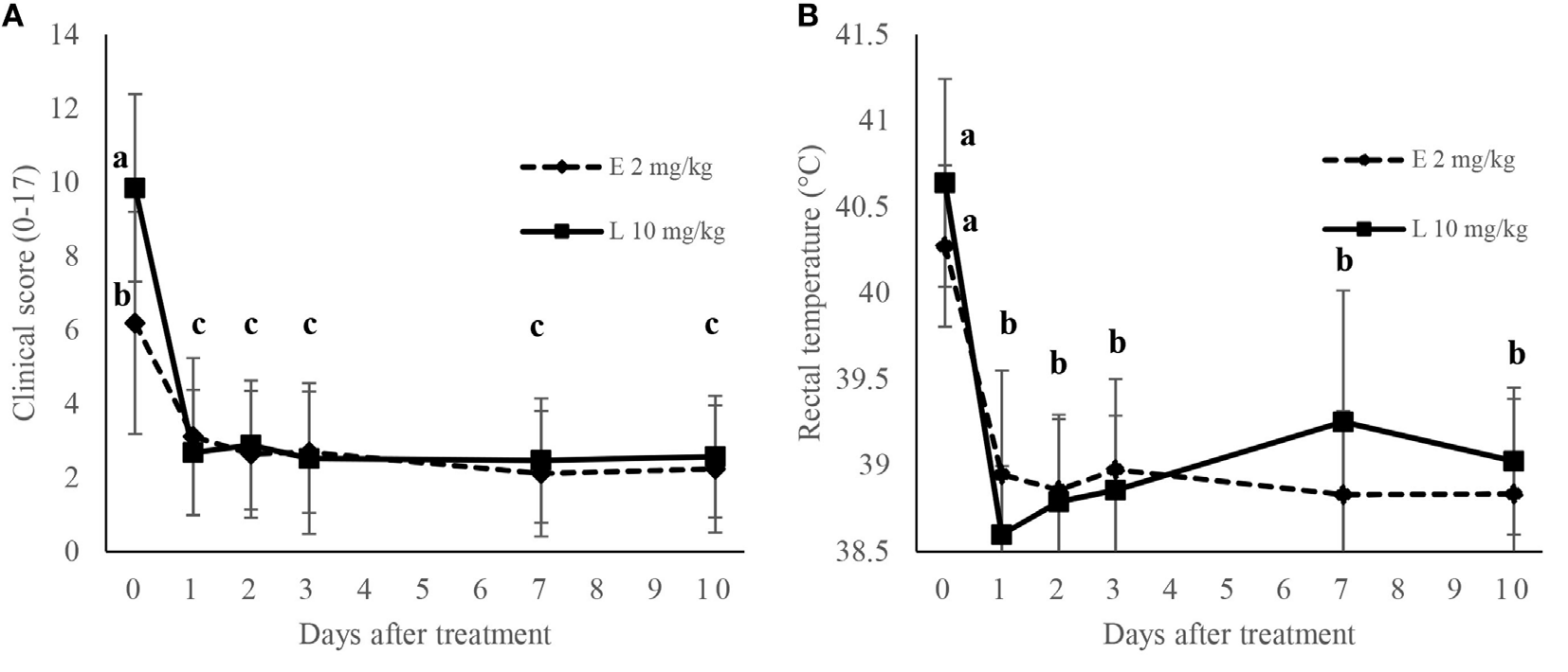
Evolution of clinical score **(A)** and rectal temperature **(B)** of young bulls treated for bovine respiratory disease with a treatment of 2 or 10 mg/kg of marbofloxacin and with tolfenamic acid. Different letters in superscripts indicate if values are statistically different (*P* < 0.05).

The evolutions of mean CS and rectal temperature values are presented in Figure 2. At the time of inclusion, mean CS and rectal temperature values were 5.9 and 40.2°C in the E2 groups, and 9.9 and 40.6°C in the L10 groups. In both groups, CS as well as rectal temperature decreased rapidly over time after treatment. 24 h after treatment, a significant decrease (*P* < 0.05) in the CS was observed in all groups. Mean CS at day 1 was 3 and 2.7 in the E2 and L10 groups, respectively, corresponding to a control CS. In the two groups, mean CS of the YB treated only once remained <4 from day 0 to day 10. No significant difference (*P* > 0.05) was observed between the groups for CS and rectal temperature from day 1 to day 10 (see Figure 2).

### Antimicrobial Consumption

Regardless of the implementation of prophylactic treatment, mean TI_UDD marbo_ per YB at risk were 16 and 43 in the E2 groups and the L10 groups, respectively, showing a significant difference between groups (*P* = 0.001). Mean TI_UDD relapse_ were significantly higher in the E2 groups compared to the L10 groups, with respective values of 25 and 3 (*P* = 0.001).

**Figure 3:**
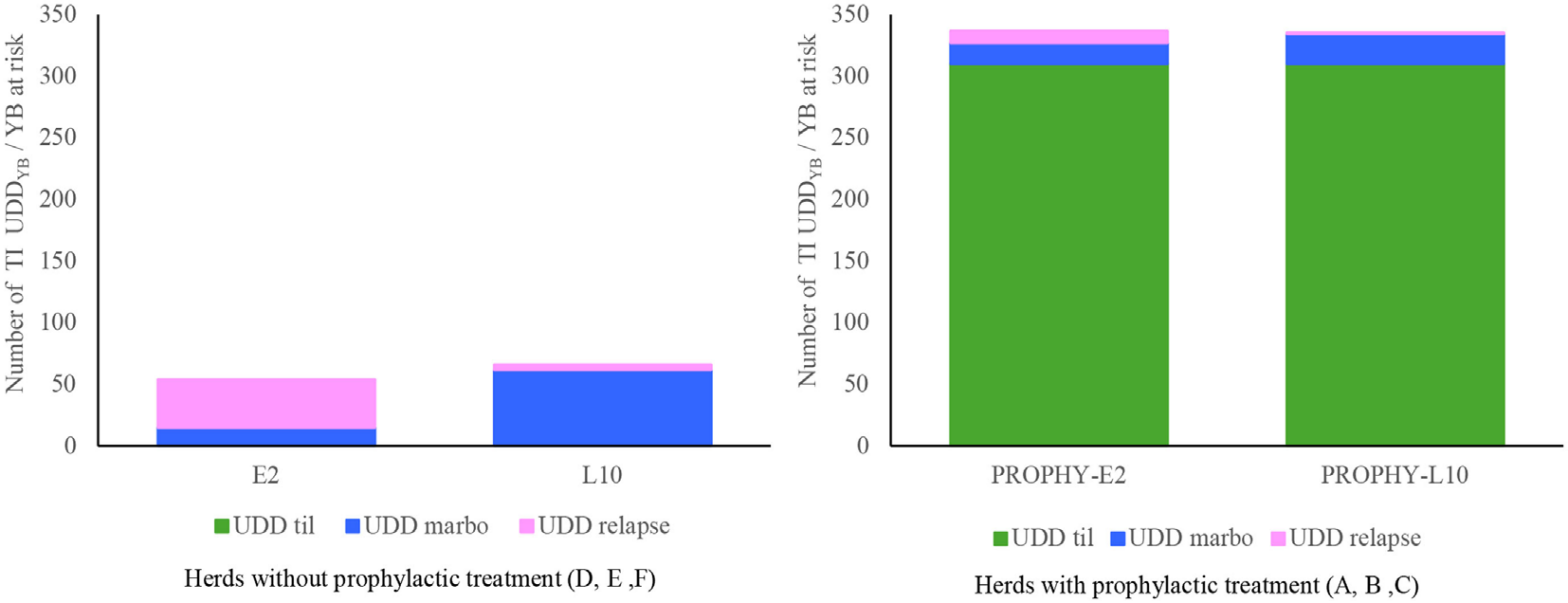
Mean antimicrobial usage (tildipirosin for prophylactic treatment, marbofloxacin as first line, tulathromycin or florfenicol as relapse), expressed in TI_UDD_, according to the prophylactic strategy implemented in the herds (absence or presence), and the therapeutic strategy [Early 2 (E2) or Late 10 (L10)]. No comparison was made between the groups with and without prophylactic treatment.

Figure 3 depicts these results and shows the relative impact of prophylactic antimicrobial treatment on global antimicrobial consumption. TI_UDD_ of prophylactic treatment represented 90% of the total TI_UDD_ in herds where a prophylactic antimicrobial treatment was implemented.

## Discussion

Our study aimed to assess the effectiveness of a protocol using an infection-stage adjusted antimicrobial regimen administered at an early stage of BRD, before the onset of clinical signs observed by farmers. We hypothesized that such regimen could be sufficient to achieve the cure of YB presenting an increase of ruminal temperature, as a proxy for onset of BRD. The fluoroquinolone antibiotic marbofloxacin was chosen to test this hypothesis in the field situation because a set of experiments previously conducted with this antibiotic in both *in vitro* and *in vivo* models have already demonstrated its validity in experimental and controlled settings.

The treatment protocol implemented in the L10 group corresponded to a traditional field situation and served as a control for comparison with the alternative protocol implemented in the E2 group. The therapy implemented in our L10 group was based upon the detection of clinical signs of BRD, in which YBs were administered marbofloxacin at the dose recommended in the SPC. The results observed in this control group (incidence rate, curative percentage, and relapse rate) were in agreement with those obtained in other field studies allowing relevant comparisons with the low dose/early treatment group of the present study (26–30).

For the early detection group, we observed that the proportion of first-line and relapse treatments were increased compared to the late detection groups, raising the question of the relevance of a therapeutic protocol combining early detection and infection-stage adjusted dose. This question of relevance can be discussed as two sub-questions: the validity of our hypothesis that a low early AMD dose would be as effective as a high late AMD dose and of the appropriateness (specificity and precision) of the body temperature to initiate an early treatment.

First, our approach was based on the assumption that the intensity of clinical signs was well correlated with the size of the bacterial load and axiomatically, that a treatment initiated before occurrence of moderate or severe clinical signs will be associated with a lower pathogen load to eliminate. It has been shown in a calf experimental model of pneumonia that the values of CS, rectal temperature, respiratory rate and lung consolidation were correlated with each other (31). While previous work on rodent pneumonia models (13, 32, 33) and our model of pneumonia in calves confirmed this relationship (18), neither the pathogen agents responsible for BRD nor the size of the inoculum at the infectious site were specified under the conditions of this field study. Although *Pasteurellaceae* are one of the most common agents of BRD in France (26), North America (6, 34, 35), and Australia (36), viruses are also often isolated and may be responsible for BRD with a similar clinical picture to what we observed.

Second, the specificity of our detection protocol based essentially on ruminal temperature monitoring to trigger an AMD treatment and the precision of its precocity compared to the occurrence of clinical symptoms are likely the most important factors generating uncertainty about the very necessity of initiating an AMD treatment, and consequently an overuse of AMD. We have to acknowledge that we are facing a lack of specific and precise tools to (i) quickly identify pathogens (viruses or bacteria) responsible for respiratory infection and (ii) assess the bacterial load at the infectious site. Hyperthermia *per se* is likely not specific enough to trigger an early infection-stage adjusted dose AMD treatment only on the YBs that should have been ineluctably treated later on with a higher dose. In the absence of an ideal diagnostic tool, we defined for body temperature a threshold based on the information observed in the experiment we conducted in calves and data from the literature to define the onset of early treatment in the field (elevation of ruminal temperature above 40.2°C for more than 12 consecutive hours, confirmed by a rectal temperature >39.7°C at the time of clinical examination (19, 20)). The rectal temperature value of 39.7°C is commonly used as the threshold value for diagnosis of abnormal temperature in YB (20, 37, 38) To ensure that YBs detected early when presenting an increased rectal temperature and no clinical signs of disease different from BRD, a physical examination was performed. Therefore, the follow-up of ruminal temperature and clinical examination before inclusion minimized the risk of including YBs not presenting BRD. Early detection of disease was confirmed by the CS and rectal temperature values at the time of inclusion, which were lower in the E2 compared to the L10 groups. However, it has been shown that clinical signs of BRD were not systematically observed in YB presenting ruminal hyperthermia (19). Hence, it is very likely that we included YB in E2 groups that subsequently would not have presented clinical signs, explaining the higher proportion of first-line treatments in the E2 groups.

Furthermore, hyperthermia and clinical signs observed in the included animals are not pathognomonic of bacterial respiratory infection (19). It is possible that some treated animals did not show any bacterial infection. As an illustration, it is commonly accepted that viral infections precede and may lead to bacterial infections; host response to a viral infection will generate an increase in temperature, and then could lead to a too early treatment. In such a case, because marbofloxacin exhibits a rapid elimination half-life (39), effective concentrations will not be maintained until the appearance of bacterial infection. Such a situation may have lead in our protocol to an increase in the proportions of relapse treatments, and also in first-line treatments. If this hypothesis is correct, an option would consist of using for early treatment an AMD product (substance or formulation) combining both inoculum-dependent pharmacodynamic properties (inoculum effect) and appropriate pharmacokinetic properties, i.e., a longer duration of action than the about 24 h in the current study.

Assuming that *Pasteurellaceae* were actually involved at the time of the early treatment, the accuracy of the reduced fluoroquinolone dose needs to be put into perspective. The selected marbofloxacin dosage for the present trial was based upon PK/PD evaluation of antimicrobial efficacy *in vitro* (40, 41) and in a mice model (42) previously published. This dosage was also successfully tested for the treatment of an experimental lung infection with *Mannheimia haemolytica* in calves (18). In this study, calves were treated with 2 mg/kg of marbofloxacin 6–10 h after inoculation of the pathogen in one group, or treated with 10 mg/kg at 36–40 h after inoculation in a second group. Efficacy of this decreased regimen administered early was found to be as good as a higher regimen at a later stage of illness and was associated with the size of the inoculum at the time of treatment. Similar results were observed in various *in vitro* and *in vivo* studies conducted with fluoroquinolones, stressing the existence of an inoculum effect (12, 13, 15, 16). In the present study, the selected dosage was the lowest dose ensuring bactericidal efficacy, assuming that early detection allowed operating on a low bacterial inoculum. In fact, we cannot rule out that at the time of initiation of our early treatment, the inoculum load to eradicate was actually higher than in our previous experiment, explaining a higher relapse rate. If this hypothesis is correct, the dose for an early treatment could be adjusted (increased) to take into account that body temperature is only a surrogate of the bacterial load to eradicate.

One of the goals of the present study was to assess the overall AMD consumption for the two tested treatment strategies. The method of evaluation of antimicrobial consumption was based on the reflection paper on collecting data of consumption developed by the European Medicines Agency (25) and on previous research conducted in livestock production (43, 44). Using indicators shared by regulatory agencies and other research units will allow comparison of drug consumption between our study and future studies using the same methodology. For each drug, UDD was assigned with the principles used by the European Medicines Agency to determine the defined daily dose for animals. In herds implementing prophylactic treatment, the proportions of first-line and relapse treatments were decreased, but without significant difference, compared with herds not implementing prophylactic treatment. These results suggest that prophylactic antimicrobial treatment decreased the proportion of clinical BRD, but might not prevent their occurrence. Furthermore, prophylactic treatments represented more than 90% of the total amount of antimicrobials. This observation suggests that rationalization of prophylactic antimicrobial treatment might significantly reduce global antimicrobial consumption without altering the control of BRD in herds. To the best of our knowledge, it is the first time that treatment incidences of UDD have been calculated in a field study with YBs; therefore, it was difficult to compare the amount of antimicrobials used with other studies.

In the present study, antimicrobial treatments were prescribed by a veterinarian following a clinical examination, and all the antimicrobial classes are approved in food producing animals. To limit the impact of antimicrobial consumption on selection of resistant bacteria, antibiotic prophylactic practices should be limited to YB at high risk of presenting BRD, and a thorough evaluation of the risk factors for BRD should be performed prior treatment initiation. Finally, the use of critically important antimicrobials, such as marbofloxacin, must be supervised by veterinarians and should be based on antimicrobial susceptibility testing, in addition to epidemiological and clinical data evaluation, to ensure their efficacy and limit the spread of AMR.

In conclusion, our results suggest that the monitoring of the evolution of body temperature of YB, as a proxy of early detection of diseased animals, is not sufficient to currently recommend treatment with an early infection-stage adjusted regimen with a rather short-action antibiotic such as marbofloxacin. Transfer in the field of the “early detection/infection stage adjusted antimicrobial treatment” remains complex and seems to depend on both PD (inoculum effect) and PK (duration of action) properties of the drug. But more importantly, it also depends on the implementation of early and precise diagnostic tools, and research should be encouraged in this direction. Specific biomarkers correlated with the bacterial load, and pathogen specific tests at the animal’s side, are of great interest to encourage veterinary precision antimicrobial therapy, i.e., optimized preventive or curative therapeutic approach (right animal, right drug, right dose, and right time) based on identification of biomarkers of disease and use of technologies of disease monitoring.

## Ethics Statement

The research protocol was compliant with the European Guideline on Good Clinical Practice (CVMP/VICH/595/98-FINAL) and has been submitted to the French National Food Safety Agency, under the reference ENR/KLD/EC-00710-0. All procedures were performed in accordance with the European Directive (2010/63/EU) and French Regulations (Decree no. 2013-118; articles R214-87 and R214-137 from Rural Code) related to the Care and Use of Laboratory Animals. However, because all the procedures involving animals were conducted in commercial farms, and therefore do not fall under the scope of the European Directive (2010/63/EU) on the protection of animals used for scientific purposes (article 1-5), ethics committees established under the French Regulations do not examine approvals of such protocols.

## Author Contributions

GL, P-LT, AB-M, and SA were responsible for the conception of the research and analyzed the data. GL, FG, and SA conducted the experiments with the help of personnel from Oniris. All the authors contributed to the redaction and approved the final manuscript.

## Conflict of Interest Statement

GL had an affiliation, and FG still has one to the commercial funder Vetoquinol. All other authors declare that the research was conducted in the absence of any commercial or financial relationships that could be construed as a potential conflict of interest.
